# Expression of Bcl-2 and p53 at the fetal-maternal interface of rhesus monkey

**DOI:** 10.1186/1477-7827-3-4

**Published:** 2005-01-14

**Authors:** Peng Wei, Xuan Jin, Xue-Sen Zhang, Zhao-Yuan Hu, Chun-Sheng Han, Yi-Xun Liu

**Affiliations:** 1 State Key Laboratory of Reproductive Biology, Institute of Zoology, Chinese Academy of Sciences, Beijing 100080, China

## Abstract

To study the apoptosis and its mechanism at the fetal-maternal interface of early gestation, localization of apoptotic cells in the implantation sites of the rhesus monkey on day 17, 19, 28 and 34 of pregnancy were first examine by using the TUNEL technique. The expression of Ki67, a molecular marker of proliferating cells, and two apoptotic proteins, B cell lymphoma/leukaemia-2 (Bcl-2) and P53, were then studied by immunohistochemistry. Apoptotic nuclei were observed mainly in the syncytiotrophoblast. Ki67 was confined almost exclusively to cytotrophoblasts. The localization of Bcl-2 protein follows that of the apoptotic nuclei and its expression level increased as the development of the placenta progressed on. P53 was detected to some extent in cytotrophoblasts and syncytiotrophoblast covering the basal feet of the anchoring villi during the late stage of placentation. Based on these observations, it might be suggested that Bcl-2 could be possible to play an interesting role in limiting degree of nuclear degradation and sustaining cell suvival in the multi-nucleated syncytiotrophoblast cells during early pregnancy, and P53 could also be essential in regulating the trophoblastic homeostasis by controlling its proliferation or apoptosis.

## Introduction

Apoptosis plays important roles in placentation and embryonic development [1]. The cells undergoing apoptosis have characteristic structural changes in the nucleus and cytoplasm. The nuclear disintegration involves DNA cleavage into oligonucleosomal length DNA fragments [2-4], and the DNA fragments can be detected by terminal deoxynucleotidyl transferase (TdT)-mediated deoxyuridine triphosphate (dUTP) nick end-labelling (TUNEL) technique. Expressions of apoptotic regulatory molecules, such as Fas, Fas ligand, P53, and the proteins of Bcl-2 family, have been reported in human placenta [5-8]. Bcl-2 and P53 are two of the key players in the apoptotic signaling cascades. Bcl-2, a proto-oncogene first discovered in human follicular lymphoma [9], is involved in the inhibition of apoptosis and the survival of a variety of cell types [10]. Bcl-2 protein is located in the membranes of endoplasmic reticulum, nuclear envelope, and mitochondria. Over-expression of Bcl-2 suppresses apoptosis by preventing the activation of caspases that carry out the process. P53 is well known as a tumor suppressor. It is a transcription factor that induces apoptosis mainly through inducing the expression of a batch of redox-related genes [11] and the down-regulating Bcl-2 [12].

The expression of Bcl-2 and P53 human placenta has been studied [1,13]. However, their cellular distribution in the implantation site at early stage of pregnancy has not been reported. Because the monkey and the human share a very similar implantation process in terms of timing, morphological changes, and cell types involved [14], we aimed, in the present study, to investigate the expression, localization of Bcl-2 and P53 in the implantation site of the rhesus monkey, in order to gain some insights to the mechanism of time-dependent apoptosis occurring at the fetal-maternal interface.

## Materials and methods

### Animals

Healthy adult male and female rhesus monkeys (*Macaca mulatta*) were purchased from the monkey colony of the Primate Research Center (PRC), Kunming Institute of Zoology (KIZ), Chinese Academy of Sciences (CAS). All experimental procedures were approved by the Animal Ethics Committees of both the Institute of Zoology and PRC. The animals were caged individually and were evaluated daily by visual examination of the perineum for menses, with the onset of menses defined as Day 1 of the menstrual cycle. Adult female monkeys with regular menstrual cycles of approximately 28 days were chosen for this study. Female monkeys on Day 11 of their menstrual cycle were caged with a male monkey of proven fertility from previous mating for 3 days. Vaginal smears were examined the next morning for the presence of sperm. The day when the smear was detected as positive for sperms was designated as Day 1 of pregnancy (D1). The presence of a conceptus was confirmed by ultrasound examination. The monkeys were anesthetized by pentobarbital sodium (3 animals each group), and the uteri were removed surgically from early villous to villous placenta stages: on D17, D19, D28 and D34 of pregnancy respectively and cut into pieces, the specimens were quickly washed in cold phosphate-buffered saline (PBS) to remove adherent blood, then placed in cold 4% paraformaldehyde fixative for 16 h at 4°C and further processed through graded dehydration, clearing and embedding in paraffin for immunohistochemistry and TUNEL assay. Part of the specimen was cryopreserved at -70°C for Western blot analysis.

### Reagents

**Figure 1:**
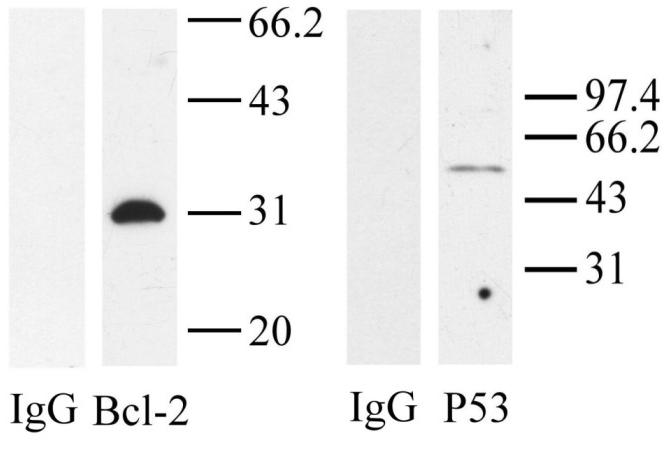
**Western blot analysis of Bcl-2 and P53 for monkey tissue obtained on D34 of pregnancy**. Specific signals of Bcl-2 and P53 proteins were detected. No band was found in the control when antibodies were replaced with normal IgG of the same concentration and origin.

Primary antibodies including rabbit anti-human P53 (SC-6243) and mouse anti-human Bcl-2 (SC-7382) were obtained from Santa Cruz Biotechnology (Santa Cruz, CA, USA). Their quality and specificity were confirmed by the result of the Western blot analysis of D34 placental proteins (Figure 1). Rabbit anti-human cytokeratin (ZA0070), mouse anti-human actin (ZA0001), and mouse anti-Ki67 (ZM0166) were purchased from Zymed laboratories (San Francisco, CA, USA). Biotin labeled secondary antibodies, alkaline phosphatase (AP) conjugated avidin, horseradish peroxidase (HRP) conjugated goat anti-rabbit IgG, HRP-conjugated horse anti-mouse IgG, and AP substrates "Vector-red" were from Vector Laboratories (Burlingame, CA, USA). Digoxigenindideoxy (DIG)-11-dUTP, TdT, blocking reagent, AP-conjugated anti-DIG antibody and 5-bromo-4-chloro-3-indoxyl phosphate/nitro-blue tetrazolium chloride (BCIP/NBT) were purchased from Roche-Boehringer-Mannheim (Mannheim, Germany). Proteinase K was purchased from Merck-Schuchardt (Darmstadt, Germany). Levamisole were purchased from Sigma-Aldrich (St Louis, MO, USA). SuperSignal^® ^West Pico substrate was from PIERCE Biotechnology (Rockford, IL, USA).

### Western blot

Western blot was done as previously described [15] with slight modifications to verify the cross-reactive specificity of the antibodies with the monkey tissue. The tissue of implantation sites on D34 of pregnancy was homogenized and the supernatant (50 μg) from centrifugation was run on a 10% SDS-PAGE gel under reduced conditions. After being transferred to the polyvinylidene difluoride membrane, individual lanes were cut and blocked with 5% nonfat milk/PBS for 1 h, followed by incubation at 20°C for 1 h with the primary antibodies (IgG, 0.2 μg/ml) in 5% milk/PBS. The membranes were washed three times, 5 min for each, in 5% milk/PBS and incubated with HRP-conjugated horse anti-mouse IgG (0.2 μg/ml, for Bcl-2) or HRP-conjugated goat anti-rabbit IgG (0.04 μg/ml, for P53) in 5% milk/PBS for 1 h respectively. The membranes were washed in PBS three times 5 min for each, followed by 10 min of incubation with SuperSignal^® ^West Pico substrate, then exposed on x-ray film. For negative controls, primary antibodies were replaced with normal IgG of the same concentration and origin.

### TUNEL

Apoptotic cells were identified by using the TUNEL technique [1,16]. The procedure was slightly modified based on Gao et al. [17] as the following. Deparaffinized and hydrated 4 μm sections were first treated with 10 μg/ml proteinase K at 37°C for 20 min, and then subjected to 3'-end-labelling of the DNA with 1 μM DIG-11-dUTP and 1 U/μl TdT at 37°C for 1 h. The sections were washes three times in Tris buffer, and incubated with blocking buffer (100 mM Tris, 150 mM NaCl, pH 7.5, and 1% blocking reagent) for 30 min at room temperature. Next, sections were incubated with the primary AP-conjugated anti-DIG antibody (1:500 in 1% blocking reagent, 100 mM Tris, and 150 mM NaCl, pH 7.5) at room temperature for 2 h, and then washed with Tris buffer. Staining was developed using the standard substrates NBT (337.5 μg/ml) and BCIP (175 μg/ml). Negative controls were similarly processed with the omission of TdT.

### Immunohistochemistry

Serial 4 μm sections of tissue were deparaffinized and rehydrated through degraded ethanol. Antigen retrieval was performed by incubating the sections in 0.01 M citrate buffer (pH 6.0) at 98°C for 20 min followed by cooling at room temperature for 20 min. Non-specific binding was blocked with 5% (v/v) normal goat serum in PBS for 1 h. The sections were incubated with primary antibodies specific for P53 (1 μg/ml), Bcl-2 (2 μg/ml) or Ki67 (2 μg/ml) respectively in 2% goat serum overnight at 4°C. Sections were then washed three times with PBS (10 min each) and incubated with biotinylated secondary antibody (2 μg/ml) at RT for 30 min. 3 × 10 min successive washes were followed by incubation with avidin-AP complex (1:200, RT, 20 min). Sections were developed with standard substrates (337.5 μg/ml NBT and 175 μg/ml BCIP) or Vector Red AP substrates according to the manufacturer's protocol after another three washes. Endogenous AP activity was inhibited by supplement of 1 mM levamisole into substrate. The sections stained with Vector Red substrates were counter-stained using haematoxylin. Sections incubated with normal IgG instead of primary antibody served as negative controls.

A double immunostaining technique using the antibodies to cytokeratin and actin was performed to localize the extravillous endovascular trophoblast cells. De-paraffinized sections were incubated with 3% H_2_O_2 _in methanol for 10 min at room temperature to quench endogenous peroxidase after antigen retrieval treatment as described above. To detect the cytokeratin signal, the sections were washed (3 × 10 min in PBS), blocked for nonspecific signals, incubated sequentially with primary anti-human cytokeratin anbibody (1 μg/ml, RT, 1 h), secondary biotinylated goat anti-rabbit IgG (2 μg/ml, RT, 30 min), and avidin-peroxidase complex (1:200, RT, 20 min), and developed with DAB substrate solution in a similar way as described above. To detect actin signal, the procedure was repeated one more time with anti-human actin antibody (1 μg/ml, RT, 2 h) as primary antibody, AP conjugated horse anti-mouse IgG (1 μg/ml, RT, 40 min) as secondary antibody, and Vector Red developing AP substrate. As a result, the trophoblast cells were labeled brown and the blood vessel wall red.

### Microscopic assessment

Placental samples from three individual monkeys for each group were analyzed. Experiments were repeated at least three times, and one representative from at least three similar results was presented. The mounted sections were examined using a Nikon microscope. For Ki67, the percentages of immunoreactive cells were assessed on at least 2000 cells in each tissue section; For TUNEL, the percentages of positive nuclei were assessed out of at least 2000 nuclei in each tissue section; For assessment of Bcl-2 staining intensity in cells of different compartments, semi-quantitative subjective scoring was performed by three blinded investigators using a 4-scale system with "-"= nil; "+/-"= weak; "+" = moderate; and "++" = strong as described by Yue et al. [18].

## Results

### Apoptosis in implantation site of early pregnancy

**Figure 2:**
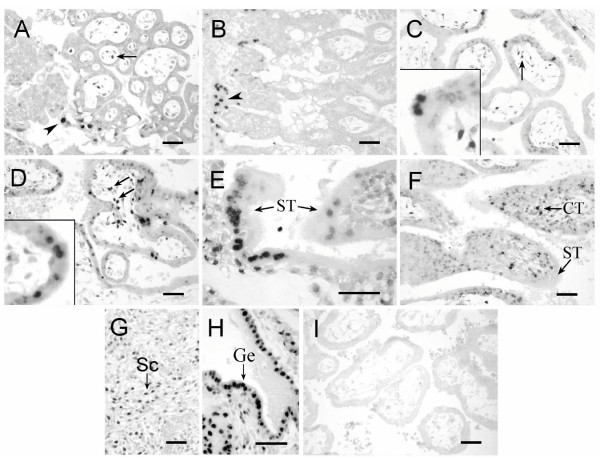
**Apoptosis detected by TUNEL at the implantation sites of the rhesus monkey on D17 (A), D19 (B), D28 (C, G) and D34 (D, E, F, H) of gestation. **Apoptotic nuclei were stained dark. Arrowhead and arrow in panel A – D indicated the nuclei of syncytiotrophoblast and villous stromal cells respectively. The insets in C and D showed the positive nuclei under a higher magnification. Note the syncytiotrophoblast layer covering the basal feet of the anchoring villi in E and the cell columns in F. G and H represent the stromal cells and glandular epithelial cells respectively in the endometrium. I was the negative control. St, syncytiotrophoblast; CT, cytotrophoblast; Vi, placental villi. Scale bars represent 50 μm.

The TUNEL technique was used to identify cell types that underwent apoptosis in the implantation site of rhesus monkey on D17, D19, D28 and D34 of pregnancy. On D17 and D19, apoptotic nuclei were observed in the syncytiotrophoblast layer covering the basal feet of the anchoring villi (Figure 2 A, B, arrowhead) and in the villous stromal cells (Figure 2 A, arrow), but not in the cytotrophoblasts. The positive nuclei in the syncytiotrophoblast was only about 0.06%. On D28 and D34, the apoptotic nuclei were present in the syncytiotrophoblast covering the villi (Figure 2 C, D), in the villous stromal cells (Figure 2 C, D, arrow), in the syncytiotrophoblast layer covering the basal feet of the anchoring villi (Figure 2 E), and in the cytotrophoblasts within the cell columns (Figure 2 F). On D28, the percentage of TUNEL-positive nuclei in the syncytiotrophoblast was 0.21%. As pregnancy progresses, the percentage increased to 0.34% on D34. In maternal compartment, a lot of apoptotic nuclei were detected in the stromal cells (Figure 2 G) and glandular epithelium (Figure 2 H).

### Proliferative activity in implantation site at early pregnancy

**Figure 3:**
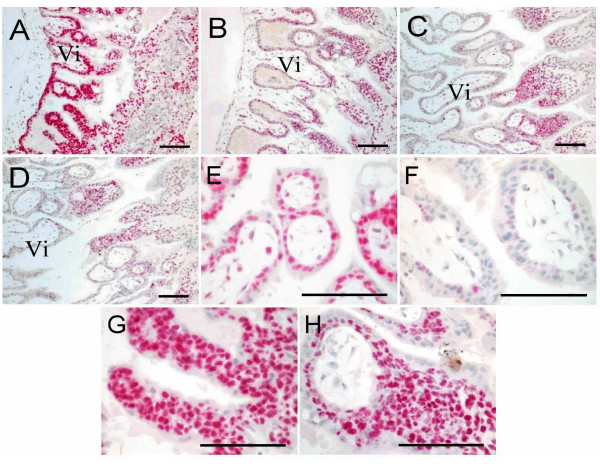
**The proliferating activity revealed by Ki-67 immunostaining at implantation sites of the rhesus monkey on D17 (A, E, G), D19 (B), D28 (C) and D34 (D, F, H) of gestation. **Panels A-D were under a lower magnification. Ki-67 protein was stained red, and nuclei blue. E and F were the placental villi under a higher magnification. G and H were the anchoring villi under a higher magnification. Vi, placental villi. ST, syncytiotrophoblast. CT, cytotrophoblast. Sc, stromal cell. En, endometrium. Scale bars represent 100 μm.

Ki67 is a protein expressed in cycling cells from G1 to M phases and is widely used as a roliferative marker (19, 20). As shown in Figure 3, Ki67 was expressed in the cytotrophoblasts and the villous stromal cells, but not in the syncytiotrophoblasts. As pregnancy progresses, the percentage of Ki67-positive cytotrophoblast cells lining the villi decreased from more than 85% on D17 to less than 25% on D34 (Panel A-D, and E, F for a higher magnification). However, the cytotrophoblasts at the proximal tip of cell columns remained highly proliferative (more than 70%) at all stages (Panel G and H).

### Bcl-2 expression in implantation site at early pregnancy

**Figure 4:**
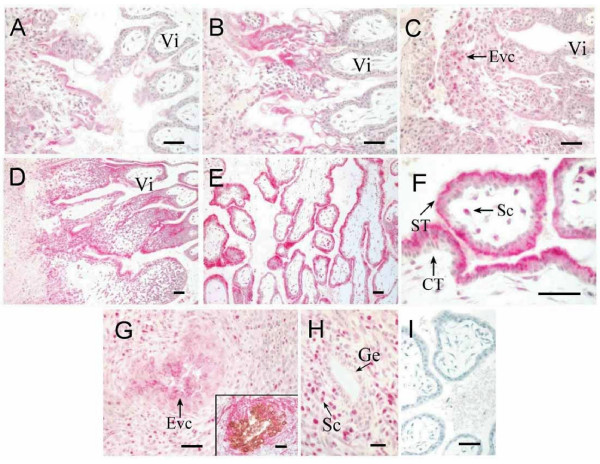
**Immunohistochemical staining for Bcl-2 at implantation sites of the rhesus monkey. **Bcl-2 staining is red, and nuclear counterstain blue. A, villous plancenta on D17. B, villous plancenta on D19. C, extravillous trophoblast cells in the basal plate of D17. D, villous plancenta on D28. E, villous plancenta on D34. F, villous plancenta on D34 under a higher magnification. G, the extravillous endovascular trophoblast cells; in the inset, the fetal origin of these cells was confirmed by anti-cytokeratin antibody (brown), and their position within the vascular wall was confirmed by anti-actin antibody staining (red). H, decidua. I, negative control. Vi, placental villi. ST, syncytiotrophoblast. CT, cytotrophoblast. Sc, stromal cells. Ge, glandular epithelium. Evc, extravillous cytotrophoblast. Scale bars represent 50 μm.

In order to study the mechanisms of the apoptosis observed at the fetal-maternal interface, the expression of Bcl-2 was investigated by using immunohistochemistry. At the early stages of placentation (D17, D19), Bcl-2 was only detected in the syncytiotrophoblast covering the cell columns (Figure 4, A and 4B) and the extravillous cytotrophoblast (Figure 4C, arrow). At the later stages (D28, D34) it was detected in all the syncytiotrophoblast (Figure 4,D and 4E), the villous stromal cells (Figure 4F, arrow), and the extravillous endovascular trophoblast cells (Figure 4G), the fetal origin of these cells were indicated by the anti-cytokeratin antibody staining (Figure 4 G inset, brown), and the vascular wall was stained by anti-actin antibody (red). The pattern of Bcl-2 expression in the syncytiotrophoblast was similar to that of the apoptotic nuclei distribution (Figure 2). In the maternal compartment, Bcl-2 could be detected in some stromal cells (Figure 4H). Notably, the cytotrophoblasts lining the villi, within the cell columns, and the glandular epithelia were negative for Bcl-2 staining. The semi-quantitative expression level of Bcl-2 in different cell types at the various stages was summarized in Table 1. A gradual increase of Bcl-2 staining was observed in the syncytiotrophoblast as gestation advances.

**Table 1:** Semi-quantitative assessment of the immunohistochemical staining of Bcl-2 in the placenta of rhesus monkey.

	D17	D19	D28	D34
Syncytiotrophoblast lining the villi	+/-	+/-	++	++
Syncytiotrophoblast covering the cell column	+	+	++	++
cytotrophoblast lining the villi	-	-	-	-
extravillous cytotrophoblast	+	+	+	+

### P53 expression in implantation site at early pregnancy

**Figure 5:**
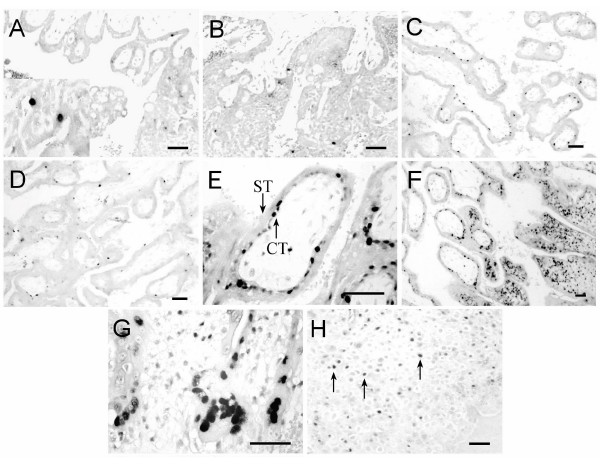
**Immunohistochemical staining for P53 at implantation sites of the rhesus monkey on D17 (A), D19 (B), D28 (C, H), and D34 (D, E, F, G) of gestation. P53 was stained dark in nuclei. **A-D were villous placenta under a lower magnification. The inset of panel A shows the staining in the syncytiotrophoblast covering the basal feet of the anchoring villi under a higher magnification. E, staining in villous placenta under a higher magnification. F, staining in cell columns. G, syncytiotrophoblast covering the basal feet of the anchoring villi under a higher magnification. H, the endometrium with arrows indicating stromal cells. ST, syncytiotrophoblast. CT, cytotrophoblast. Scale bars represent 50 μm.

The expression profile of P53 was also acquired by using the immunohistochemistry. On D17 and D19, the expression of P53 was only confined to a small number of nuclei in the syncytiotrophoblast (Figure 5,A,B). On D28 and D34, its expression was observed not only in the syncytiotrophoblast (Figure 5C,D) but also in the nuclei of cytotrophoblasts lining the villi (Figure 5E) and within proximal tip of cell columns (Figure 5F) where a proliferative activity was high as indicated by Ki67 staining (Figure 3). Clustered P53-positive nuclei were seen in the syncytiotrophoblast covering the basal feet of the anchoring villi (Figure 5G), coincident well with the strong apoptosis detected by TUNEL (Figure 2E). P53 was also expressed in some stromal cells (Figure 5H) of the uterine endometrium.

## Discussion

For the first time in present study, we investigated the expression of Bcl-2 and P53 in relation to apoptosis at the fetal-maternal interface of rhesus monkey at the very early stages (D17-D34) of gestation. Villous trophoblasts consist of cytotrophoblasts and syncytiotrophoblast. While cytotrophoblasts possess a brisk mitotic activity during the first trimester of gestation in human, the syncytiotrophoblast is incapable of cell division despite of a metabolic activity [1]. This fact implies that cell proliferation is differently regulated in these two cell types. The reports on the type of trophoblast cells undergoing apoptosis in the first trimester are controversial [1,21,22]. Our results further cleared that the apoptotic nuclei were distributed mainly in the syncytiotrophoblast at the early stages and in the cytotrophoblasts within the cell columns at later stages of pregnancy.

**Figure 6:**
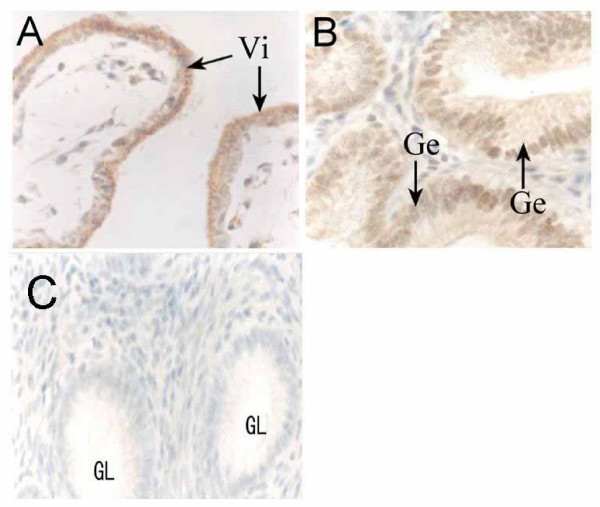
**Immunohistochemical staining for Bax at implantation sites of the rhesus monkey on D28. **Bax staining is brown, and nuclear counterstain blue. A, villous plancenta, positive staining was found in all the cells. B, endometrium, glandular epithelium was positive for Bax staining. Vi, placental villi. Ge, glandular epithelium.

In our previous study, Bax expression was found at the Fetal-Maternal Interface of Rhesus Monkey [17]. Bax is a Bcl-2 family member that promotes cell death susceptibility, possibly by countering the effect of Bcl-2 on cell survival through heterodimer interaction. Bax to Bcl-2 "rheostat" may be a critical factor in regulating apoptosis in multiplicate tissues. As shown in Figure 6, Bax was found expressed in the placenta and glandular epithelium of endometrium and all kinds of cells in placental villi, and no obvious change was observed between different time points from D17 to D34 in placental villi. Therefore, we speculated that Bcl-2 may play a more important role on controlling the apoptosis in placental villi. The diffusive expression of Bcl-2 in syncytiotrophoblast obtained from the first trimester human placenta has been reported recently [7,23-25]. Our observation on the Bcl-2 expression in syncytiotrophoblast at later stages (D28-D34) agreed well with these data. As shown in this study, although Bcl-2 was expressed, apoptotic nuclei still exsisted in the same region. This phenomenon implies that the expression of Bcl-2 is not sufficient to completely inhibit the apoptosis in the syncytiotrophoblast. Therefore, the role of Bcl-2 here becomes an interesting question. Multiple nuclei sharing the same cytoplasm is a morphological characteristic of syncytiotrophoblast. In such cells, the apoptotic signal may be transmitted from one nuclear to another, and cause a spontaneous abortion. Therefore, the number of nuclei undergoing apoptosis in the syncytiotrophoblast should be limited by some mechanism in order to ensure normal embryo development in normal pregnancy [1]. We speculate that Bcl-2 may be included in this mechanism. The major role of apoptosis-associated Bcl-2 expression in the syncytiotrophoblast might be to limit the nuclear degradation to a special area and inhibit the spread of cell apoptosis signals to the other nuclei sharing the same cytoplasm, thus sustain cell survival in these multi-nucleated cells. Toki et al has also suggested that Bcl-2 might play a major role in avoiding the possible excessive nuclear degradation in syncytiotrophoblast [26]. Further studies, however, are needed to prove this speculation. The immunostaining for Bcl-2 was also detected in part of the extravillous and endovascular cytotrophoblast in our study. These subtypes of cytotrophoblast lost the capacity of proliferation (Ki-67-negative), but they did not undergo apoptosis (negative in TUNEL assay). Therefore, we hypothesize that Bcl-2 may also participate in regulation of the extravillous trophoblast apoptosis by stimulating the cellular survival.

P53 was partly identified in some nuclei of the syncytiotrophoblast with the same position of apoptotic nuclei, in the basal feet of the anchoring villi in particular, but it is not clear whether the P53 was co-localized with the apoptotic signals. Activation of P53 in some cell types leads to either the cessation of cell growth or apoptosis [27]. Therefore, P53 protein might be related to cell cycle arrest or apoptosis in syncytiotrophoblast during early stage of placentation. Low level of P53 staining was detected in the cytotrophoblasts during the earlier stages of gestation (D17 and D19). However, at the later stages (D28 and D34), the expression was observed predominantly in the nuclei of cytotrophoblasts. The presence of P53 in cytotrophoblast in the primate was consistent with that observed in the human first trimester placenta [8]. Indeed, the TUNEL staining showed that the apoptosis seldom happened in the cytotrophoblast, with the exception of cytotrophoblast at proximal tip of cell columns during later stages of placentation (D28, D34) where a high proliferative activity and P53 expression were detected. This finding supports the hypothesis that a physiological upregulation of the P53 tumour suppressor gene might be a mechanism for controlling excessive trophoblastic proliferation in normal placentation [26,28].

It is known that early pregnancy is unique in its methods of cell proliferation control, the existing data suggest that some growth factors and transcription factors from the embryo and endometrium, such as CSF-1, VEGF, and transcription factors of the helix-loop-helix family, provide at least part of this control [29]. In addition, other studies found maternal age and some diseases, such as diabetes can also influence the apoptotic and proliferative activities in trophoblast cells [30,31]. Further investigations are required to uncover which endocrine event regulates the expression of Bcl-2 and P53.
